# Supplementary material for: Identification of QTL under Brassinosteroid-Combined Cold Treatment at Seedling Stage in Rice Using Genotyping-by-Sequencing (GBS)
Source: Plants (Basel). 2022 Sep 5;11(17):2324. doi: 10.3390/plants11172324 (PMC9460439; doi:10.3390/plants11172324)
Supplement: Supplementary file 1 [file plants-11-02324-s001.zip › plants-1878916-supplementary.pdf]

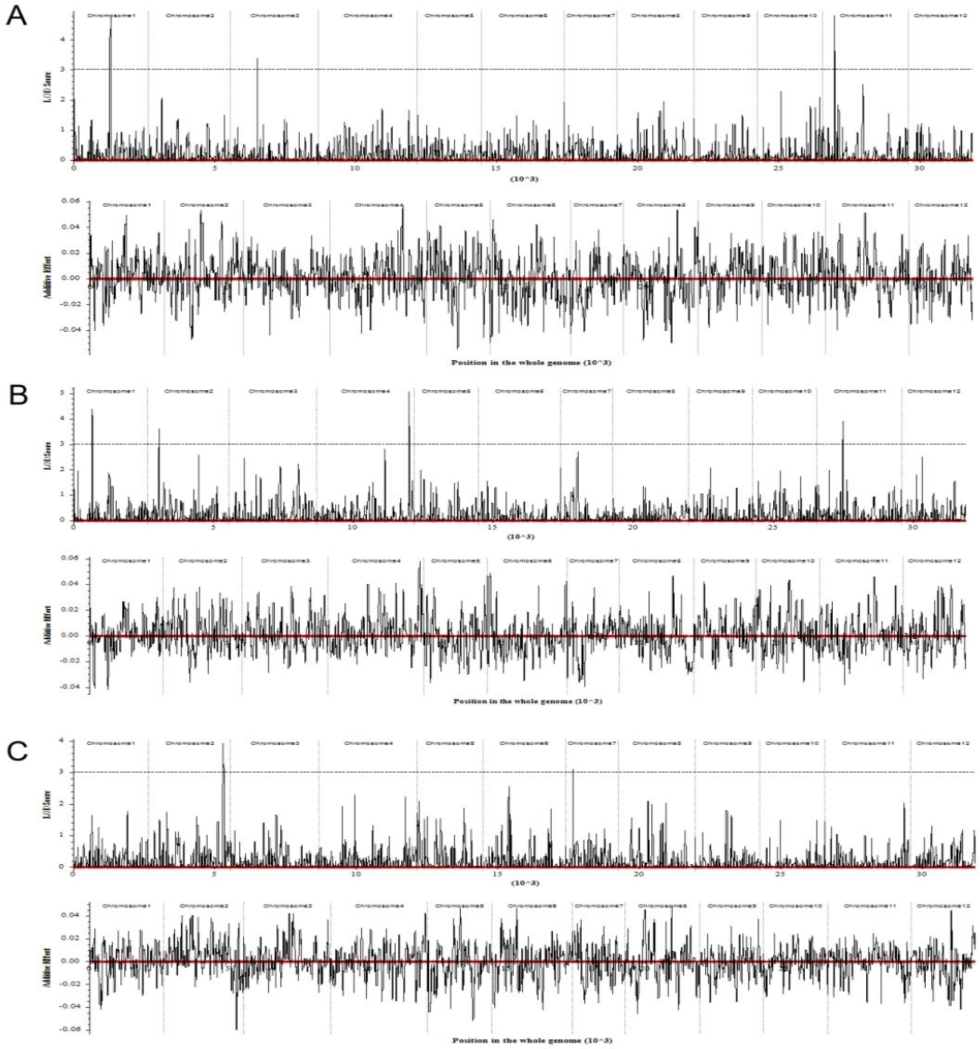

Figure S1. QTL mapping based on seedling shoot length (SSL). (**A**) Cold treatment; (**B**) Normal temperature condition were used as the control; (**C**) Cold treatment condition were used as the control.

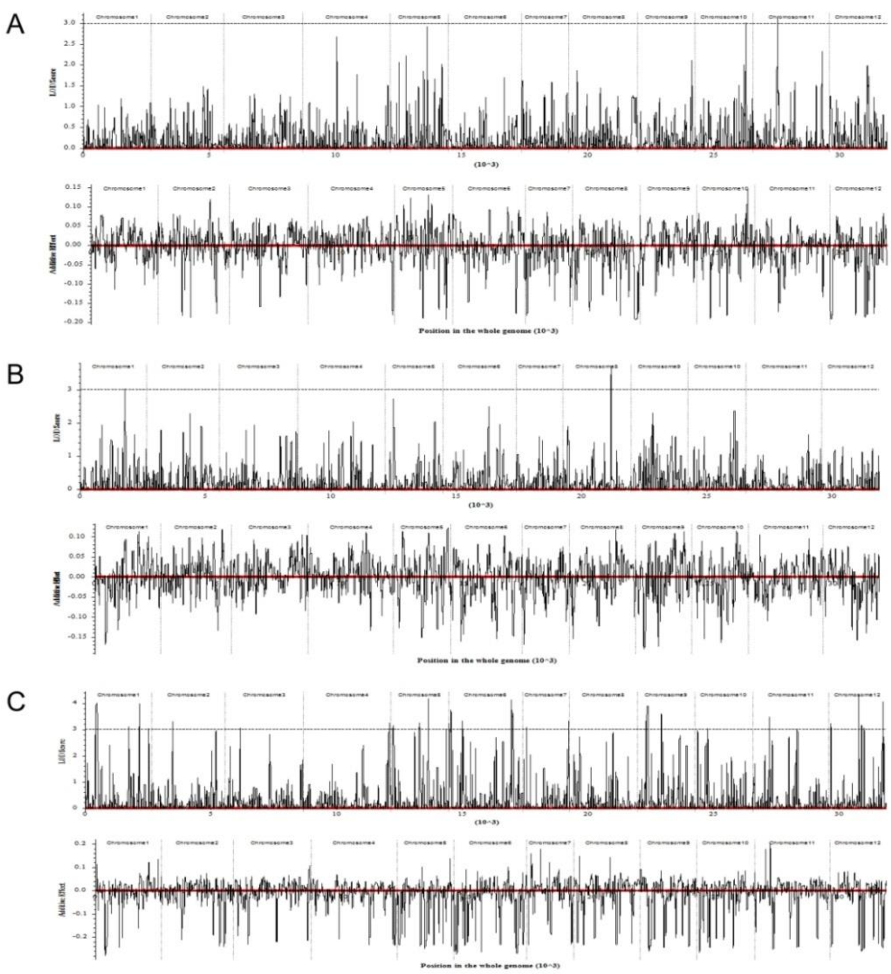

Figure S2. QTL mapping based on seedling root length (SRL). (**A**) Cold treatment; (**B**) Normal temperature condition were used as the control; (**C**) Cold treatment condition were used as the control.

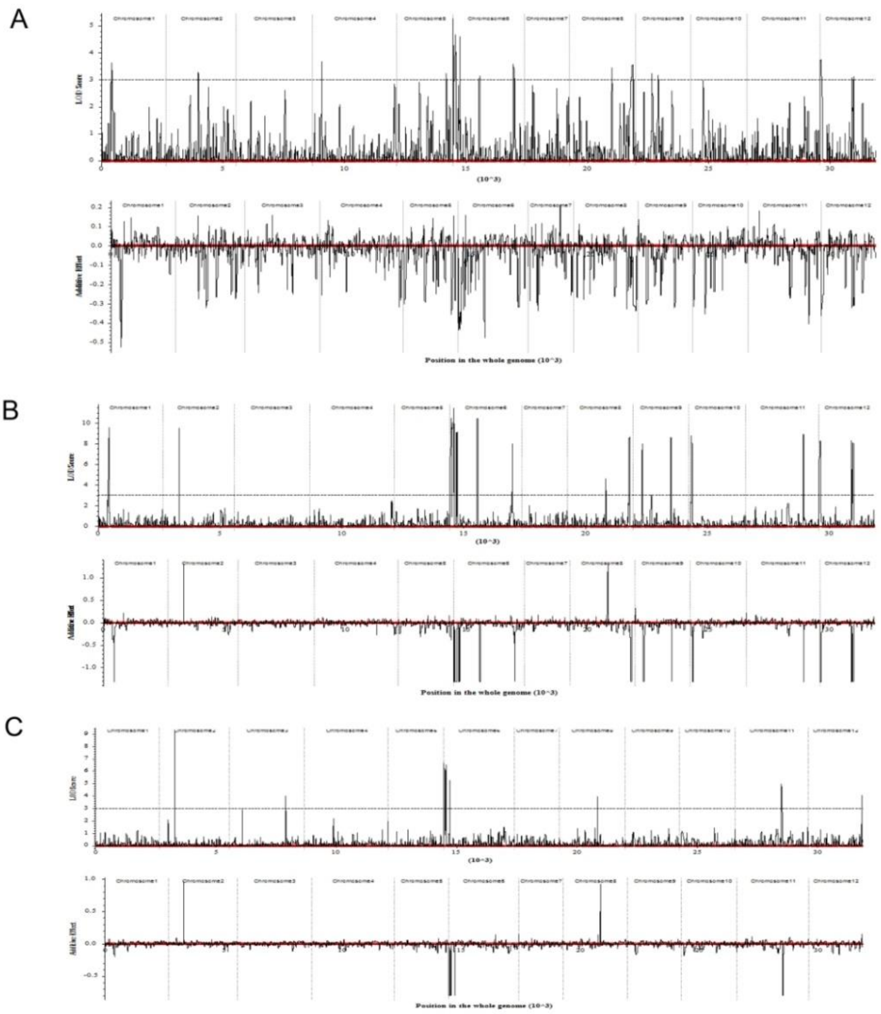

Figure S3. QTL mapping based on seedling dry weight (SDW). (**A**) Cold treatment; (**B**) Normal temperature condition were used as the control; (**C**) Cold treatment condition were used as the control.

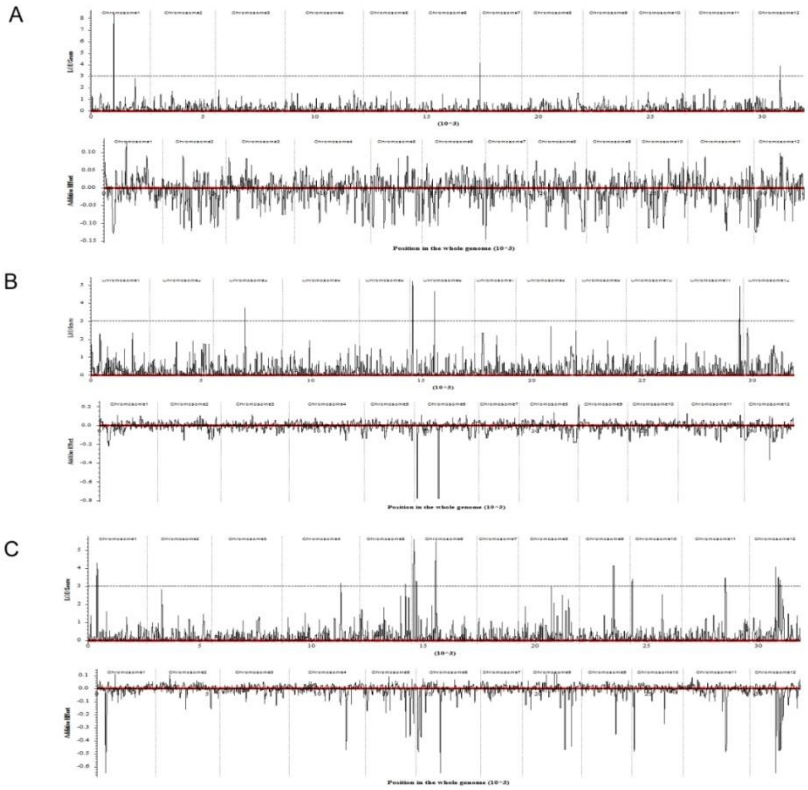

Figure S4. QTL mapping based on seedling wet weight (SWW). (**A**) Cold treatment; (**B**) Normal temperature condition were used as the control; (**C**) Cold treatment condition were used as the control.

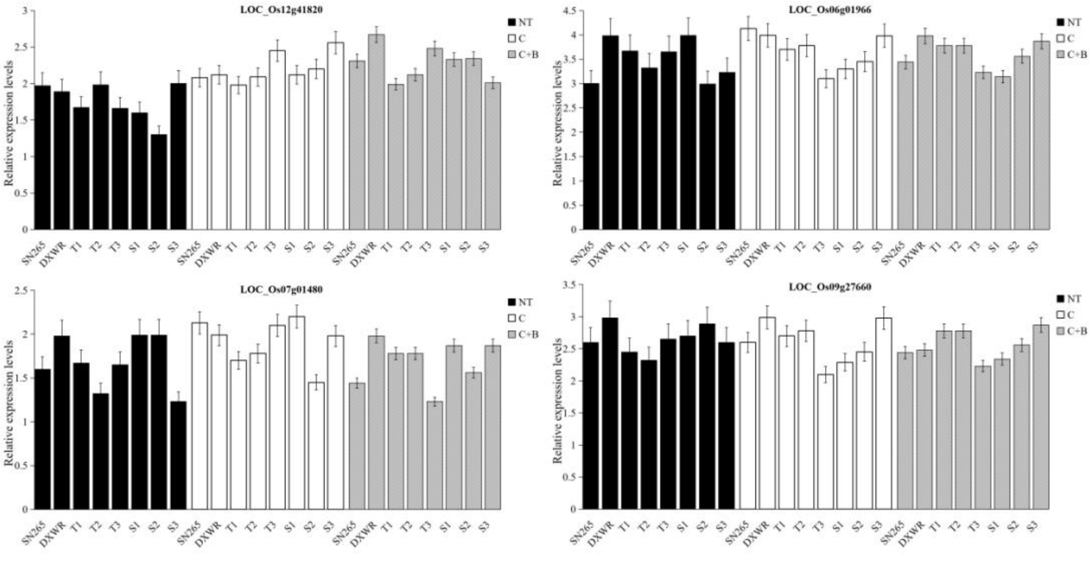

Figure S5. Expression patterns of some candidate genes (LOC_Os12g41820, LOC_Os06g01966, LOC_Os07g01480, and LOC_Os09g27660). NT, normal temperature; C, Cold stress; C+B, BR combined cold treatment.

Table S1. Details of primers used for this study.

Gene ID or Name	Forward primer (5′-3′)	Reverse primer (5′-3′)
LOC_Os01g40260	CACTCAAAGCAGCACAACCC	CGATCTTGCTGCCGTTCTTG

LOC_Os11g45740	GCACAACATTTGCCTGGGAG	GGCGAGGCATCCTAGGTAC
LOC_Os05g39380	AAATTCCACCATTTGCGCCC	GGCCCCTCAAACCTACTCAC
LOC_Os07g05805	GAGAACAACAGAGGCGGGA	GGCTTACATCCCTTGCGGTA
LOC_Os12g41820	CCAGGCGTATACAGTGCACA	CCGCACAAACAAAGCTCGA
LOC_Os06g44750	GCCGGAAACAAGGACCTGTA	TTGAGGATGCTGTCGACGTC
LOC_Os06g01966	TTGCTGCACTCCTAAGGGTG	AACTTGGTGTGTCAAGGCCA
LOC_Os07g08440	GGACTTGGTCCCCAACACAA	CCCAGCCTGCTCATACTCAG
LOC_Os07g01480	CGCCTCAAGTCCACCTTCAT	GGATGATCTTGGTGTCGGCA
LOC_Os09g27660	TCATGTGAAAGGCGCCATCT	GCTCACCTGACTCAAAAGC
LOC_Os03g50885(*actin*)	TCGAGCATGGTATCGTCAGC	GGATGGCGAGTACATAGCA

Table S2. Summary of all candidate genes.

Condition	QTL	Chr	Locus name	Gene coordinates	Gene product
Cold	qSSL1-2	1	LOC_Os01g40260	22731943-22733237	OsWRKY77 - Superfamily of TFs having WRKY and zinc finger domains
BRs+Cold	qSWW11-2	11	LOC_Os11g45740	27670321-27673334	MYB family transcription factor
BRs+Cold	qSRL9-2	9	LOC_Os09g04504	2396780-2400181	Iron and phosphorus responsive genes
BRs+Cold	qSDW3-1	3	LOC_Os03g50090	28568746-28571308	Iron and phosphorus responsive genes
BRs+Cold	qSSL11-2	11	LOC_Os11g04104	1657863-1668362	Major facilitator superfamily antiporter
old	qSSL11-1	11	LOC_Os11g11960	6640922 - 6638262	disease resistance protein RPM1, putative, expressed
Cold	qSSL11-1	11	LOC_Os11g11990	6655976 - 6663781	NB-ARC domain containing protein, expressed
BRs+C-N	qSSL1-1	1	LOC_Os01g21670	12161768 - 12157455	plant protein of unknown function domain containing protein, expressed
BRs+C-N	qSSL1-1	1	LOC_Os01g21710	12184857 - 12186827	plant protein of unknown function domain containing protein, expressed
BRs+C-N	qSSL1-1	1	LOC_Os01g21820	12248213 - 12235262	preprotein translocase subunit secA, chloroplast precursor, putative, expressed
BRs+C-N	qSSL1-1	1	LOC_Os01g21850	12263046 - 12258138	protein transport protein-related, putative, expressed
BRs+C-N	qSSL7-1	7	LOC_Os07g05805	23483968..23485531	OsBZR1, Transcription factor, Brassinosteroid (BR)-regulated growth response
BRs+C-N	qSSL1-1	1	LOC_Os01g21860	12264911 - 12269085	AGAP010768-PA, putative, expressed
BRs+C-C	qSRL5-3	5	LOC_Os05g39380	23101671 - 23104153	zinc finger, C3HC4 type domain containing protein, expressed
BRs+C-C	qSRL5-3	5	LOC_Os05g39390	23109898 - 23104197	outer membrane protein, OMP85 family protein, expressed
BRs+C-C	qSRL12-6	12	LOC_Os12g41680	25800944 - 25806553	No apical meristem protein, putative, expressed
BRs+C-C	qSRL12-6	12	LOC_Os12g41690	25812631 - 25807626	membrane associated DUF588 domain containing protein, putative, expressed
BRs+C-C	qSRL12-6	12	LOC_Os12g41700	25818874 - 25815277	LSD1 zinc finger domain containing protein, expressed
BRs+C-C	qSRL12-6	12	LOC_Os12g41710	25824699 - 25820749	Protein kinase domain containing protein, expressed
BRs+C-C	qSRL12-6	12	LOC_Os12g41715	25832900 - 25828364	DEAD-box ATP-dependent RNA helicase, putative, expressed

BRs+C-C	qSRL12-6	12	LOC_Os12g41720	25840479 - 25838564	PLA IIIA/PLP7, putative, expressed
BRs+C-C	qSRL12-6	12	LOC_Os12g41780	25882977 - 25885922	glycosyl transferase family 17 protein, putative, expressed
BRs+C-C	qSRL12-6	12	LOC_Os12g41790	25886211 - 25887243	DEFL3 - Defensin and Defensin-like DEFL family, expressed
BRs+C-C	qSRL12-6	12	LOC_Os12g41800	25892992 - 25888923	VTC2, putative, expressed
BRs+C-C	qSRL12-6	12	LOC_Os12g41820	25907573 - 25901456	heat shock protein DnaJ, putative, expressed
BRs+C-C	qSRL12-6	12	LOC_Os12g41830	25914643 - 25911188	Methyltransferase small domain containing protein, expressed
BRs+C-C	qSRL12-6	12	LOC_Os12g41840	25917832 - 25916322	protein transport protein Sec61 subunit alpha, putative, expressed
BRs+C-C	qSRL12-6	12	LOC_Os12g41860	25927353 - 25920639	START domain containing protein, expressed
Cold/BRs+C-N	qSDW1-3/4	1	LOC_Os01g17070	9816783 - 9822750	Putative Deg protease homologue
Cold/BRs+C-N	qSDW6-18/17	6	LOC_Os06g44750	27025437 - 27029339	AP2 domain containing protein, expressed
Cold/BRs+C-N	qSDW9-2/3	9	LOC_Os09g10850	5940418 - 5935331	meiotic coiled-coil protein 7, putative, expressed
Cold/BRs+C-N	qSDW9-2/3	9	LOC_Os09g10840	5918077 - 5929775	transcription factor, putative, expressed
Cold/BRs+C-N	qSDW12-5/6	12	LOC_Os12g33962	20514770 - 20515834	uncharacterized tatC-like protein ymf16, putative, expressed
Cold/BRs+C-N	qSDW12-5/6	12	LOC_Os12g33958	20503266 - 20509254	NADH-ubiquinone oxidoreductase 49 kDa subunit, putative, expressed
BRs+C-N/ BRs+C-C	qSDW6-9/10	6	LOC_Os06g01966	543057 - 545780	auxin-induced protein 5NG4,putative, expressed
BRs+C-N	qSDW9-1	9	LOC_Os09g04624	2458920 - 2474678	GDSL-like lipase/acylhydrolase, putative, expressed
BRs+C-N	qSDW9-1	9	LOC_Os09g04670	2493538 - 2489758	DAG protein, chloroplast precursor, putative, expressed
BRs+C-N	qSDW9-1	9	LOC_Os09g04710	2501667 - 2504421	GDSL-like lipase/acylhydrolase, putative, expressed
BRs+C-N	qSDW9-1	9	LOC_Os09g04720	2508294 - 2505261	SWIB/MDM2 domain containing protein, expressed
BRs+C-N	qSDW9-1	9	LOC_Os09g04730	2517277 - 2512441	dehydrogenase/reductase SDR family member 2, putative, expressed
BRs+C-N	qSDW9-5	9	LOC_Os09g27620	16786041 - 16788255	PHD-finger domain containing protein, putative, expressed
BRs+C-N	qSDW9-5	9	LOC_Os09g27650	16822234 - 16825686	ZOS9-14 - C2H2 zinc finger protein, expressed
BRs+C-N	qSDW9-5	9	LOC_Os09g27660	16829409 - 16837307	OsFBO21 - F-box and other domain containing protein, expressed
BRs+C-N	qSDW10-1	10	LOC_Os10g04590	2185226 - 2188547	OsFBX358 - F-box domain containing protein, expressed
BRs+C-N	qSDW10-1	10	LOC_Os10g04600	2191954 - 2194603	OsFBX359 - F-box domain containing protein, expressed
BRs+C-N	qSDW10-1	10	LOC_Os10g04610	2196559 - 2198967	OsFBX360 - F-box domain containing protein, expressed
BRs+C-N	qSDW10-1	10	LOC_Os10g04620	2201493 - 2206735	OsPOP20 - Putative Prolyl Oligopeptidase homologue, expressed
BRs+C-N	qSDW10-1	10	LOC_Os10g04674	2237611 - 2226986	disease resistance protein RPM1, putative, expressed
BRs+C-N	qSDW10-1	10	LOC_Os10g04700	2245973 - 2243002	OsFBX361 - F-box domain containing protein, expressed
BRs+C-N	qSDW10-1	10	LOC_Os10g04720	2252231 - 2249380	TKL_IRAK_DUF26-la.5 - DUF26 kinases have homology to DUF26 containing loci, expressed
BRs+C-N	qSDW10-1	10	LOC_Os10g04730	2257490 - 2262626	TKL_IRAK_DUF26-la.6 - DUF26 kinases have homology to DUF26 containing loci, expressed
BRs+C-N	qSDW10-1	10	LOC_Os10g04750	2279444 - 2278550	OsFBX362 - F-box domain containing protein, expressed
BRs+C-N	qSDW10-1	10	LOC_Os10g04780	2300571 - 2299438	OsFBX363 - F-box domain containing protein, expressed
Cold	qSWW7-1	7	LOC_Os07g01310	191044 - 197084	glutamate receptor, putative, expressed
Cold	qSWW7-1	7	LOC_Os07g01340	213778 - 219544	gibberellin 2-beta-dioxygenase 7, putative, expressed
Cold	qSWW7-1	7	LOC_Os07g01370	235903 - 237446	peroxidase precursor, putative, expressed
Cold	qSWW7-1	7	LOC_Os07g01380	239515 - 240945	peroxidase precursor, putative, expressed
Cold	qSWW7-1	7	LOC_Os07g01400	252818 - 253867	peroxidase precursor, putative, expressed

Cold	qSWW7-1	7	LOC_Os07g01410	259712 - 262744	peroxidase precursor, putative, expressed
old	qSWW7-1	7	LOC_Os07g01420	264525 - 265650	peroxidase precursor, putative, expressed
Cold	qSWW7-1	7	LOC_Os07g01440	291032 - 292026	plastocyanin-like domain containing protein, putative, expressed
Cold	qSWW7-1	7	LOC_Os07g01450	294566 - 292640	DNA directed RNA polymerase, 7 kDa subunit domain containing protein, expressed
Cold	qSWW7-1	7	LOC_Os07g01470	302864 - 303349	circumsporozoite protein precursor, putative, expressed
Cold	qSWW7-1	7	LOC_Os07g01480	306009 - 307555	oxygen evolving enhancer protein 3 domain containing protein, expressed
Cold	qSWW7-1	7	LOC_Os07g01490	309744 - 306009	kinesin motor domain containing protein, putative, expressed
BRs+C-C	qSWW1-2	1	LOC_Os01g17070	9816783 - 9822750	OsDegp1 - Putative Deg protease homologue, expressed
BRs+C-C	qSWW9-1	9	LOC_Os09g27620	16786041 - 16788255	PHD-finger domain containing protein, putative, expressed
BRs+C-C	qSWW9-1	9	LOC_Os09g27650	16822234 - 16825686	ZOS9-14 - C2H2 zinc finger protein, expressed
BRs+C-C	qSWW9-1	9	LOC_Os09g27660	16829409 - 16837307	OsFBO21 - F-box and other domain containing protein, expressed
BRs+C-N	qSSL1-1	1	LOC_Os01g21840	12250987 - 12257719	transposon protein, putative, unclassified, expressed
BRs+C-C	qSRL12-6	12	LOC_Os12g41750	25869905 - 25867093	transposon protein, putative, unclassified, expressed
Cold/BRs+C-N	qSDW1-3/4	1	LOC_Os01g17080	9825970 - 9823099	transposon protein, putative, unclassified, expressed
Cold/BRs+C-N	qSDW6-18/17	6	LOC_Os06g44800	27067208 - 27063980	transposon protein, putative, Ac/Ds sub-class, expressed
BRs+C-N	qSDW10-1	10	LOC_Os10g04760	2282724 - 2280969	transposon protein, putative, CACTA, En/Spm sub-class, expressed
Cold	qSWW7-1	7	LOC_Os07g01390	242462 - 248622	transposon protein, putative, CACTA, En/Spm sub-class, expressed
BRs+C-C	qSWW1-2	1	LOC_Os01g17080	9825970 - 9823099	transposon protein, putative, unclassified, expressed
BRs+C-N	qSSL1-1	1	LOC_Os01g21690	12166733 - 12170415	retrotransposon, putative, centromere-specific, expressed
BRs+C-N	qSSL1-1	1	LOC_Os01g21720	12189343 - 12187950	retrotransposon protein, putative, Ty3-gypsy subclass
BRs+C-N	qSSL1-1	1	LOC_Os01g21760	12209585 - 12210813	retrotransposon protein, putative, unclassified
BRs+C-C	qSRL12-6	12	LOC_Os12g41730	25846192 - 25852257	retrotransposon protein, putative, unclassified, expressed
BRs+C-C	qSRL12-6	12	LOC_Os12g41740	25862884 - 25862018	retrotransposon protein, putative, unclassified
Cold/BRs+C-N	qSDW6-1/2	6	LOC_Os06g01060	48391 - 40328	retrotransposon protein, putative, Ty3-gypsy subclass, expressed
Cold/BRs+C-N	qSDW9-2/3	9	LOC_Os09g10900	5986513 - 5989914	retrotransposon protein, putative, unclassified, expressed
Cold/BRs+C-N	qSDW9-2/3	9	LOC_Os09g10890	5971976 - 5977219	retrotransposon protein, putative, unclassified, expressed
Cold/BRs+C-N	qSDW9-2/3	9	LOC_Os09g10870	5954108 - 5958902	retrotransposon protein, putative, unclassified, expressed
BRs+C-N	qSDW9-1	9	LOC_Os09g04520	2406717 - 2410208	retrotransposon protein, putative, Ty3-gypsy subclass, expressed
BRs+C-N	qSDW9-1	9	LOC_Os09g04530	2415665 - 2419187	retrotransposon protein, putative, Ty3-gypsy subclass, expressed
BRs+C-N	qSDW9-1	9	LOC_Os09g04540	2422444 - 2419520	retrotransposon protein, putative, unclassified, expressed
BRs+C-N	qSDW9-1	9	LOC_Os09g04550	2425674 - 2430409	retrotransposon protein, putative, unclassified, expressed
BRs+C-N	qSDW9-1	9	LOC_Os09g04560	2434843 - 2434379	retrotransposon protein, putative, unclassified, expressed
BRs+C-N	qSDW9-1	9	LOC_Os09g04570	2436804 - 2439580	retrotransposon protein, putative, unclassified
BRs+C-N	qSDW9-1	9	LOC_Os09g04580	2440452 - 2444776	retrotransposon protein, putative, unclassified
BRs+C-N	qSDW9-1	9	LOC_Os09g04590	2447267 - 2446629	retrotransposon protein, putative, unclassified, expressed

BRs+C-N	qSDW9-1	9	LOC_Os09g04610	2455233 - 2457153	retrotransposon protein, putative, Ty3-gypsy subclass, expressed
BRs+C-N	qSDW9-1	9	LOC_Os09g04660	2481910 - 2485993	retrotransposon protein, putative, unclassified, expressed
BRs+C-N	qSDW9-1	9	LOC_Os09g04740	2527005 - 2522416	retrotransposon protein, putative, Ty3-gypsy subclass, expressed
BRs+C-N	qSDW9-1	9	LOC_Os09g04750	2534111 - 2534927	retrotransposon protein, putative, Ty3-gypsy subclass, expressed
BRs+C-N	qSDW9-1	9	LOC_Os09g04780	2544661 - 2544897	retrotransposon protein, putative, unclassified
BRs+C-N	qSDW9-5	9	LOC_Os09g27640	16801495 - 16799952	retrotransposon protein, putative, unclassified
BRs+C-N	qSDW10-1	10	LOC_Os10g04640	2215376 - 2219718	retrotransposon protein, putative, unclassified, expressed
BRs+C-N	qSDW10-1	10	LOC_Os10g04740	2271236 - 2265971	retrotransposon protein, putative, unclassified, expressed
BRs+C-N	qSDW10-1	10	LOC_Os10g04810	2322519 - 2319464	retrotransposon protein, putative, Ty3-gypsy subclass
Cold	qSWW7-1	7	LOC_Os07g01330	208790 - 208326	retrotransposon protein, putative, unclassified
Cold	qSWW7-1	7	LOC_Os07g01350	226028 - 226378	retrotransposon protein, putative, unclassified, expresse
Cold	qSWW7-1	7	LOC_Os07g01360	232835 - 230588	retrotransposon protein, putative, unclassified, expressed
Cold	qSWW7-1	7	LOC_Os07g01430	272067 - 278026	retrotransposon protein, putative, Ty3-gypsy subclass, expressed
Cold	qSWW7-1	7	LOC_Os07g01500	313665 - 319435	retrotransposon protein, putative, unclassified, expressed
Cold	qSWW7-1	7	LOC_Os07g01510	323322 - 320065	retrotransposon protein, putative, unclassified, expressed
BRs+C-C	qSWW9-1	9	LOC_Os09g27640	16801495 - 16799952	retrotransposon protein, putative, unclassified
BRs+C-N	qSSL1-1	1	LOC_Os01g21700	12176157 - 12176726	hypothetical protein
BRs+C-N	qSSL1-1	1	LOC_Os01g21730	12199004 - 12198238	hypothetical protein
BRs+C-N	qSSL1-1	1	LOC_Os01g21800	12231162 - 12231556	hypothetical protein
BRs+C-N	qSSL1-1	1	LOC_Os01g21830	12249408 - 12248605	hypothetical protein
BRs+C-N	qSDW9-1	9	LOC_Os09g04700	2500313 - 2500021	hypothetical protein
BRs+C-N	qSDW9-1	9	LOC_Os09g04760	2535764 - 2535378	hypothetical protein
BRs+C-N	qSDW9-1	9	LOC_Os09g04770	2540148 - 2539390	hypothetical protein
Cold	qSSL11-1	11	LOC_Os11g11970	6646433 - 6647140	expressed protein
Cold	qSSL11-1	11	LOC_Os11g11980	6648986 - 6650344	expressed protein
BRs+C-N	qSSL1-1	1	LOC_Os01g21660	12154685 - 12156630	expressed protein
BRs+C-N	qSSL1-1	1	LOC_Os01g21680	12164080 - 12163770	expressed protein
BRs+C-N	qSSL1-1	1	LOC_Os01g21740	12204078 - 12203402	expressed protein
BRs+C-N	qSSL1-1	1	LOC_Os01g21750	12205888 - 12205547	expressed protein
BRs+C-N	qSSL1-1	1	LOC_Os01g21780	12220348 - 12224299	expressed protein
BRs+C-N	qSSL1-1	1	LOC_Os01g21790	12225766 - 12225123	expressed protein
BRs+C-N	qSSL1-1	1	LOC_Os01g21810	12232535 - 12234767	expressed protein
BRs+C-N	qSSL1-1	1	LOC_Os01g21870	12270402 - 12270037	expressed protein
BRs+C-C	qSRL12-6	12	LOC_Os12g41760	25875554 - 25873568	expressed protein
BRs+C-C	qSRL12-6	12	LOC_Os12g41770	25878380 - 25877950	expressed protein
BRs+C-C	qSRL12-6	12	LOC_Os12g41810	25898041 - 25894317	expressed protein
BRs+C-C	qSRL12-6	12	LOC_Os12g41850	25918563 - 25920483	expressed protein
Cold/BRs+C-N	qSDW1-3/4	1	LOC_Os01g17060	9796174 - 9793959	expressed protein
Cold/BRs+C-N	qSDW1-3/4	1	LOC_Os01g17065	9810633 - 9809343	expressed protein
Cold/BRs+C-N	qSDW6-1/2	6	LOC_Os06g01050	33589 - 37124	expressed protein
Cold/BRs+C-N	qSDW6-18/17	6	LOC_Os06g44790	27062657 - 27059070	expressed protein
Cold/BRs+C-N	qSDW6-18/17	6	LOC_Os06g44780	27054106 - 27054779	expressed protein
Cold/BRs+C-N	qSDW6-18/17	6	LOC_Os06g44770	27047926 - 27046712	expressed protein
Cold/BRs+C-N	qSDW6-18/17	6	LOC_Os06g44760	27043124 - 27045828	expressed protein
Cold/BRs+C-N	qSDW9-2/3	9	LOC_Os09g10880	5961154 - 5966211	expressed protein
Cold/BRs+C-N	qSDW9-2/3	9	LOC_Os09g10865	5950962 - 5951439	expressed protein
Cold/BRs+C-N	qSDW9-2/3	9	LOC_Os09g10860	5943847 - 5943545	expressed protein
Cold/BRs+C-N/ BRs+C-C	qSDW6-5/6/4	6	LOC_Os06g01070	50099 - 50641	expressed protein
BRs+C-N	qSDW9-1	9	LOC_Os09g04504	2400181 - 2396780	expressed protein
BRs+C-N	qSDW9-1	9	LOC_Os09g04508	2405236 - 2401956	expressed protein
BRs+C-N	qSDW9-1	9	LOC_Os09g04600	2450436 - 2450046	expressed protein

BRs+C-N	qSDW9-1	9	LOC_Os09g04650	2479042 - 2478779	expressed protein
BRs+C-N	qSDW9-1	9	LOC_Os09g04690	2497809 - 2497167	expressed protein
BRs+C-N	qSDW10-1	10	LOC_Os10g04625	2209029 - 2208448	expressed protein
BRs+C-N	qSDW10-1	10	LOC_Os10g04630	2212018 - 2212907	expressed protein
BRs+C-N	qSDW10-1	10	LOC_Os10g04660	2220769 - 2224867	expressed protein
BRs+C-N	qSDW10-1	10	LOC_Os10g04690	2240843 - 2239218	expressed protein
BRs+C-N	qSDW10-1	10	LOC_Os10g04710	2247890 - 2248524	expressed protein
BRs+C-N	qSDW10-1	10	LOC_Os10g04770	2292768 - 2289182	expressed protein
BRs+C-N	qSDW10-1	10	LOC_Os10g04800	2317293 - 2317584	expressed protein
BRs+C-C	qSDW11-1	11	LOC_Os11g39920	23791977 - 23795809	expressed protein
BRs+C-N/ BRs+C-C	qSWW6-2	6	LOC_Os06g01070	50099 - 50641	expressed protein
Cold	qSWW7-1	7	LOC_Os07g01320	199857 - 195763	expressed protein
Cold	qSWW7-1	7	LOC_Os07g01460	299704 - 301426	expressed protein
BRs+C-C	qSWW1-2	1	LOC_Os01g17060	9796174 - 9793959	expressed protein
BRs+C-C	qSWW1-2	1	LOC_Os01g17065	9810633 - 9809343	expressed protein
BRs+C-C	qSWW9-1	9	LOC_Os09g27610	16781152 - 16784471	expressed protein

BRs+C-N, the data under normal temperature condition were used as the control; BRs+C-C, the data under cold treatment condition were used as the control. .
